# Evaluation of Antineoplasic Activity of Zingiber Officinale Essential Oil in the Colorectal Region of Wistar Rats

**DOI:** 10.31557/APJCP.2020.21.7.2141

**Published:** 2020-07

**Authors:** Daniel Augusto Nunes de Lima, Brenda Barroso Pelegrini, Felipe Alexandre Alves Uechi, Raíssa Coracini Varago, Bruno Bueno Pimenta, Alice Maria de Souza Kaneshima, Edilson Nobuyoshi Kaneshima, Paola da Costa Souza, Raíssa Bocchi Pedroso, Thaís Gomes Verzignassi Silveira, Tânia Cristina Alexandrino Becker

**Affiliations:** 1 *Department of Basic Health Sciences, State University of Maringa, UEM, Brazil.*; 2 *Department of Medicine, State University of Maringa, Brazil. *; 3 *Department of Clinical Analysis and Biomedicine, State University of Maringa, Brazil. *; 4 *General Pathology Laboratory, State University of Maringa, Department of Basic Health Sciences, Brazil. *

**Keywords:** Colorectal neoplasms, aberrant crypt focus, ginger – zingiber officinale, essential oil, chemopreventive effect

## Abstract

**Introduction:**

Aberrant Crypt (AC) and Aberrant Crypt Focus (ACF) are considered pre-neoplasic lesions, ranging from hyperplasia to different degrees of dysplasia in the colon. This work aimed to evaluate and quantify the chemopreventive activity of Zingiber officinale essential oil in the colorectal region of Wistar rats.

**Materials and Methods:**

We extracted the essential oil from ginger rhizomes and carried out ACF induction, in rats, with 1.2 Dimethylhydrazine (DMH) at a 20 mg/kg dose. The experimental groups were GI (negative control); GII (positive induction control); GIII (DMH + essential oil); GIV (DMH +5-Florouracil) and GV (essential oil). The histological techniques used were methylene blue, hematoxylin-eosin (HE) dyeing, and immunohistochemistry (IHQ).

**Results:**

The major essential oil compounds were citral (17.25%), δ-citral (10.25%), camphene (9.55%), α-zingiberene (7.57%), nerol (6.37%) and plelandrene (6.83%). For the presence of AC or ACF, we did not observe them in GI and GV, while in GII and GIII, they were observed, in high values, in both regions, but only in the distal region, there was a significant difference between them. For GIV, for both regions, there were significant lower numbers of AC when compared to GIII. As observed, with HE, there were hyperplastic and dysplastic ACF in the proximal and distal portions of the colon. For IHQ analyses, there were positively PCNA antibody marked cells in all experimental groups. Yet, there was no significant correlation of mitotic index among them. Moreover, the results of GIII compared to GIV were very similar.

**Conclusion:**

In this sense, the Zingiber officinale essential oil has good antioxidant potential because it presents a mixture of monoterpene and sesquiterpene compounds. Thus, it is able to develop a chemoprotective effect, as it presented similar results to the standard drug, showing cell proliferation control.

## Introduction

Colorectal cancer (CRC) is the second most frequent cause of death in Western countries. It is also listed as the third most common cancer and the fourth leading cause of death (Eser et al., 2018; Moati et al., 2018). In Brazil, mortality rates for this type of cancer have increased in southern and south-eastern regions, which have similar characteristics to industrialized countries (Dutra et al., 2018). 

Several risk factors (genetic and environmental) contribute to the increased incidence of various types of neoplasms in humans, including CRC. This cancer is substantially influenced by environmental factors that interfere with the oxidative state of cells. Moreover, diets rich in red and processed meat (sausages) as well as alcoholism, smoking, obesity, old age and physical inactivity (sedentariness) are seen as important risk factors for CRC (Valadão and Castro, 2018; Mendonça et al., 2012). 

The development of colon cancer is a multi-stage process ranging from discrete microscopic formations, such as pre-neoplastic lesions, to malignant tumours (BIRD, 1995). 1.2-Dimethylhydrazine (DMH) is a complete carcinogen, often used in experimental models for inducing carcinogenesis in rodent colon (Bird and Good, 2000; Ganaie et al., 2019). Stimuli capable of causing lesion in the intestinal mucosa are able to alter the cellular differentiation of colonic crypts, leading to the formation of aberrant crypts (AC) and aberrant crypt focus (ACF) which project into the intestinal lumen. These are distinguished from normal crypts by their darker coloration, larger size, larger pericryptal zone and elliptical shape, whose surface is easily visualized by light microscopy stained with methylene blue (Tanaka, 2009; Pan et al., 2011).

According to Raju (2008), two types of ACF can be identified in both rodents and humans: hyperplasics and dysplasics. ACF can originate from independent initiation events and present distinct morphological characteristics, ranging from hyperplasia to different degrees of dysplasia (cellular atypias involving tumour progression) (Cheng and Lai, 2003). The proliferating nuclear cell antigen (PCNA) has been classified as an important marker of cell proliferation in the colon sections (Eisenberg and Koifman, 2001).

Ginger, which belongs to the Zingiberaceae family and the Zingiber species, is known both for its medicinal properties and as a condiment in several tropical and subtropical regions of the world (Blanco et al., 2016). The rhizomes of ginger are used for the extraction of essential oil (GEO) that consists mainly of sesquiterpene hydrocarbons and monoterpenes, found in smaller quantities and coming in many forms: cineole, linalol, sapwood, neral, citral and geraniol (Voon et al., 2012).

In addition, other active elements such as terpenes and oleoresin are present in ginger (Voon et al., 2012). However, these components may vary according to its place of origin and if fresh or dry (Fernandes et al., 2016). Among the pharmacological properties of the GEOs, their antioxidant action is of great interest because it inhibits or retards the appearance of cancer cells, besides delaying cell aging by neutralizing the excess of free radicals in the body (Barreiros et al., 2006; Togar et al., 2014). 

In this sense, studies that characterize the distribution and quantification of AC in the colon can be used to evaluate agents for its chemopreventive and/or antitumor activity. Therefore, this study aimed to evaluate and quantify the chemoprotective activity of Zingiber officinale essential oil (GEO) by analysing pre-neoplastic lesions in Wistar rats colon.

## Materials and Methods

*Methodology*

Obtaining the essential oil from Zingiber officinale

Fresh Zingiber officinale rhizomes were acquired in Maringá city, Paraná state, Brazil (latitude: -23, 4273, longitude: -51, 9375), cultivated by rural producers in Marialva city, Paraná state, Brazil (latitude: -23, 4855, longitude: -51, 7927). The essential oil was extracted by hydrodistillation using a Clevenger apparatus according to the methods described by the European Pharmacopoeia (Council of Europe, 1997). The extraction was performed with 200g of ginger rhizomes/ 500 mL of distilled water, boiled for 180 min. Anhydrous sodium sulphate (Labsynth®, Diadema, Brazil) was added to the extracted oil to remove residual moisture. The oil was stored at 4°C and protected from light (Singh et al., 2008). For a better characterization of the oil obtained, a sample was analysed by gas chromatography with mass spectrometer (CG-MS) in the Research Support Center Complex (ComCap/UEM).

*Chemical characterization of the GEO components*

Gas chromatography (GC) analysis was performed by a GC apparatus (Thermo Electron Corporation Focus), with DB-5 capillary column (30 m × 0.32 mm 0.50 µm); temperature of 60°C (1 min) to 180°C at 3°C/min rate; injection and detection temperature of 220°C with a1:10division ratio. He as a drag gas, with a flow rate of 1.0 mL/min. The injected volume was 1µL diluted in acetone (1:10). The GC/MS analysis was performed with a quadruple mass spectrometer (Thermo Electron Corporation, model DSQ II) operating at 70eV. Identification of the individual components was based on comparison GC retention rates with reference to the n-alkane C_8_H_18_-C_20_H_42 _series (Sigma, USA) in a DB-5 column. By comparison, authentic standard mass spectra acquired from Sigma-Aldrich, and mass spectra described in literature data was done (Adams, 2007). 

*Pharmaceutical Drugs used*

We used DMH (1.2-dimethylhydrazine) at a 20 mg/Kg concentration (Rodrigues et al., 2002) as a neoplastic inducing drug (1.5% EDTA in phosphate buffer solution, pH 6.5). As standard treatment, we used the commercial drug Fauldfluor^®^ (LIBBS) whose active ingredient is 5-Florouracil (5-FU), used as CRC conventional treatment, at a 35mg/kg concentration (dose adequacy for Wistar rats according to Moghadamtousi and collaborators (2015)). Both drugs were administered intraperitoneally (IP) once a week for every 10 weeks. The GEO obtained was orally administered to all animals of the experimental treatment group at a 300 mg/kg concentration (33μl/100g animal weight) (Sharma et al., 1994).

*Experimental design*

Twenty-eight male Wistar rats were used, obtained from the Maringa State University´s (UEM) Central Bioterium, weighing between 150 - 200 grams. The animals were kept in the sectorial UEM´s Pharmacology and Therapeutics Department (LIFIN) Bioterium, in collective cages containing three animals per cage, lined with wood sawdust, receiving special rats feed (Nuvital CR1) and water ad libitum. The animals were housed with controlled temperature (20ºC+2ºC), humidity (60-70%) and light and dark light cycles (12/12hs). They were divided into five experimental groups with six animals per group (except negative control group with n=04). The rats from all groups were euthanized at the end of the 10^th^ week.

Group I (GI) - Negative control = animals without induction and without treatment, inoculated only with the DMH dilution solution.

Group II (GII) - Positive control induction = animals with DMH induction. 

Group III (GIII) - Experimental treatment group = induction with DMH + treatment with the GEO (33μl/100g animal weight) (Sharma et al., 1994).

Group IV (GIV) - Standard treatment group = induction with DMH +5-FU

Group V (GV) - Control experimental treatment = animals treated only the GEO.

Twelve hours before euthanasia, the animals were food deprived by transferring the groups to metabolic cages. After that, they received thiopental, intravenously, at a 30 mg/kg dose. We removed their large intestines along their entire length, washed in saline solution, sectioned in the mesenteric line, and stretched on Styrofoam plates and fixed with paraformaldehyde (4%) for six hours. Subsequently, we measured all the tissue extension and stored it in 70% ethanol.

*Histopathological analysis and quantification of AC and ACF*

The colonic mucosa was stained with methylene blue 1.0% (Dias et al., 2010). The AC and ACF were identified and quantified according to criteria established by Bird (1987), tabulated as isolated AC and ACF with 02 to 03, 04 to 09 or more than 10 as AC/focus. After this stage, tissue samples from both regions (proximal and distal) were dihydrated in increasing alcoholic solution baths, diaphanized in xylol and included in paraffin. The paraffinized blocks were sectioned in a semi-automatic microtome with 4-5 mm fragments, and attached to histological slides. On each slide, 04 or 05 samples from each region were placed and submitted to successive baths of xylol and alcohol, and stained with Hematoxylin Eosin (HE). On average, 20 fields/cut were analysed under 200 times magnification and the ACF were histologically classified as hyperplasic and dysplasic according to Yoshimi et al., (2004).

*AC and ACF Immunohistochemical Analysis*

Pre-silanized slides received tissue samples from each experimental group and were submitted to the Immunohistochemical Reaction (IHQ) protocol, composed of the stages: a) blocking of endogenous peroxidase with a solution of hydrogen peroxide (3.5%) in methanol; b) antigenic recovery with 10 mM citrate buffer pH 6.0; c) blocking of non-specific binding with BSA (bovine albumin) and Donkey Serum d) incubation with PCNA primary Antibody (monoclonal PCNA PC-10 - Invitrogen) (overnight); e) washing with PBS and incubation with Super Picture™ Polymer Detection Kit -(Invitrogen); f) incubation with DAB chromogen (diaminobenzidine) (Invitrogen) and g) counter staining with Hematoxylin.

*Quantitative Analysis of the PCNA Expression*

To determine the quantitative expression of the PCNA we used a computerized system consisting of a light microscope (Opticam microscopy technology). The images were captured along the entire histological section through its coupled camera (Opticam Lopt 14003).The positive and negative count of cells for PCNA expression was performed in at least 20 perpendicular crypts of normal appearance, increased 400 times in the most intensely stained areas. After scanning the images, 100 cells of the mucous epithelium were counted in random fields (Scopa et al., 2003) with the aid of Image Processing and Analysis – Image Pro plus software. The PCNA was considered positive in the cells of the colon’s crypts that showed a very evident dark brown nuclear staining. The protein expression index was calculated using the following formula: [(number of cells marked with PCNA) / (total number of cells)] x100, thus obtaining the Mitotic Index (MI) of each group (Yamashita et al., 1994).

*Statistical analysis*

The experiments were described as mean ± standard deviation of the number of crypts foci and aberrant crypt foci per animal in each group. The data obtained were analyzed by Oneway ANOVA variance, followed by Turkey’s Test to compare the means. The significance level adopted was 5%.

*Ethical aspects*

UEM´s Ethical Committee of animal experiments Conduction – Registry n 8281191017, approved the experiments. All procedures in this study were conducted in accordance to the 3Rs rules, following the ARRIVE guidelines (www.nc3rs.org.uk/ARRIVE). The UEM´s Pharmacology and Therapeutics (LIFIN) sectorial Bioterium is in accordance to the country current legislation (Law n 11.794/2008).

## Results

**Figure 1:**
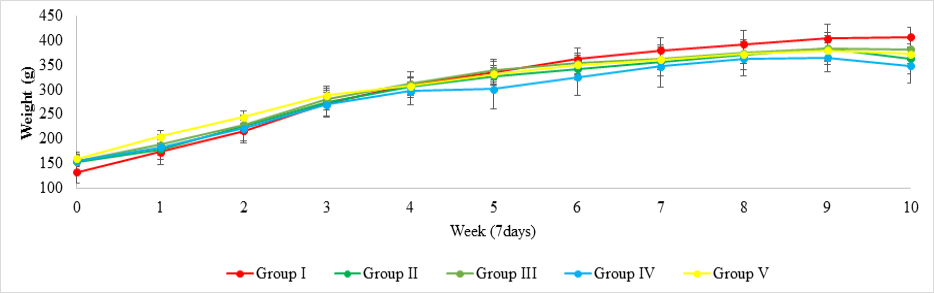
Animals Weight Assessment. The graph expresses the body weight in grams of the animals that compose the groups, monitored weekly and, the results presented, represents the average animals’ weight per group per week

The essential oil of Zingiber officinale Roscoe (ginger) produced and used in our experimental groups had in total, 17 chemical components (Table 01). The majoritarian compounds found were citral (17.25%), δ-citral (10.25%), camphene (9.55%), α-zingiberene (7.57%) and nerol, plelandrene (6.37% and 6.83%, respectively). All experimental groups in this study presented a positive variation in relation to the weight gain of the animals, both in the control and treatment groups (Figure 1). Food and water intake, controlled for 30 days, showed no significant differences between the groups (data not shown). 

**Figure 2.**
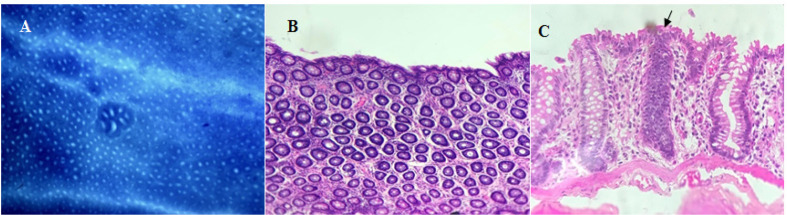
Representative Images of the Study's Histological in Experimental Groups (GII, GIII and GIV). A, Colonic mucosa stained with methylene blue, representing a topographic view of an ACF with seven AC. The AC presents an irregular luminal opening and a thicker epithelial lining compared to normal adjacent crypts (light microscopy - 4x objective). B/C: Colonic mucosa stained with H E representing a topographic view; B, hyperplastic crypts, with abundant glandular formations. The luminal opening of ACF was slightly elevated from the normal mucosa, presenting several elongated crypt layers; C, dysplastic AC (black arrow) presented cells with marked cytoplasmic basophilia; high nucleus-cytoplasm ratio and loss of cell polarity in several degrees, nuclear stratification; and reduction in mucin production

The histological analyses performed by methylene blue staining are expressed in Table 02, by mean ± standard deviation. We observed that in the control groups (I and V) there was no development of AC or ACF in any tissue sample. In GII (induction control) high values of proximal and distal AC and ACF were observed in all animals. The amount of AC in the proximal region of GIII was also quite high, but did not differ significantly from GII, whereas in the distal region we found a noteworthy reduction. The number of isolated AC in the proximal and distal regions of GIV was notably lower in relation to GIII. Figure 2A illustrates an ACF with seven AC. The results observed in this sense showed that in GIII there was a reduction with remarkable difference from GII in the ACF, with more than four crypts/focus on the proximal region. However, in the distal region, we observed considerable reduction in foci with more than two and more than four crypts. In foci with more than ten crypts, there was no difference for any region. In relation to GIV (standard treatment) the values of ACF with two to three crypts/focus in the proximal region had significant difference, but in the other classes (above four and ten crypts/focus), we observed values without statistical difference.

In addition to these parameters, with the use of HE staining, we evaluated and quantified the presence of ACF with hyperplasia and dysplasia (Figures 2B and 2C, respectively) in the proximal and distal portions of the colon. We observed that in the sections of GI and GV there were not AC or ACF outbreaks. On the other hand, GII, GIII and GIV presented a large amount of hyperplasic and dysplastic crypts both in the proximal and distal portions. GII presented significantly higher values of hyperplasic and dysplastic ACF than the standard and experimental treatment groups. 

**Figure 3.**
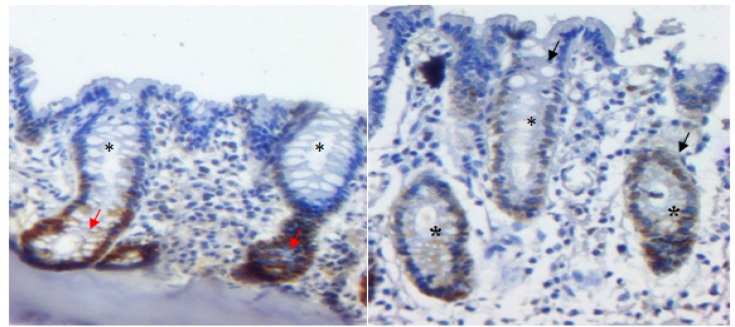
Colon Photomicrographs Marked with PCNA and Developed with DAB. Crypt cells in the basal region of the crypt (red arrow) and crypt apex in experimental groups (GII, GIII and GIV) (black arrow) with marked expression of PCNA, presence of nuclear stratification and reduced mucin production (*).

By means of immunohistochemical analyses, our results showed positively marked cells for PCNA in all experimental groups (Figure 3), both in the basal region (proliferative area of crypts) and in apical regions. In GII there were more positive cells than in the other groups. The mitotic index (MI) in GII was 19.12% in the proximal portion and 27.97% in the distal portion. Yet, there was no significant correlation of MI among the experimental groups. On the other hand, when we compared them with GIII results (9.31% (proximal) and 10.44% (distal)) we noticed a considerable reduction. Moreover, the MI results of GIII in relation to the MI of GIV (7.27% (proximal) and 11.59% (distal)) were very similar. 

The groups that were not submitted to induction with the carcinogen (GI and GV), showed much lower MI compared to the induced groups (2.09% and 1.88% (proximal) and 3.51% and 3.82% (distal)), respectively. In this sense, we can affirm that DMH is a great inducer of cell proliferation. In our study, no tumours were observed in any experimental group.

**Table 1:** Chemical Composition of Ginger Essential Oil (Zingiber officinale) Analyzed by Gas chromatography

Components	trA	n	m	Dn	trn	trm	IKA	%
α-Pinene	6.45	9	10	1	5.54	8.64	929.35	3.39
Camphene	6.96	9	10	1	5.54	8.64	945.81	9.55
D-Limonene	9.63	9	10	1	5.54	8.64	1031.94	1.99
β-Phellandrene	9.70	10	11	1	8.64	12.58	1026.90	6.83
Eucalyptol	9.75	10	11	1	8.64	12.58	1028.17	6.68
Borneol	15.52	11	12	1	12.58	16.91	1167.90	1.08
p-mentol-1-en-8-ol	16.52	11	12	1	12.58	16.91	1190.99	1.27
δ-Citronellol	17.90	12	13	1	16.91	21.31	1222.50	0,28
δ-Citral	18.40	12	13	1	16.91	21.31	1233.86	10.25
Trans-Geraniol	18.93	12	13	1	16.91	21.31	1245.91	4.47
Citral	19.70	12	13	1	16.91	21.31	1263.41	17.25
NerolAcetate	24.47	13	14	1	21.31	25.61	1373.49	6.37
Benzene	28.71	14	15	1	25.61	29.72	1475.43	6.18
(-)Zingiberene	29.29	14	15	1	25.61	29.72	1489.54	7.57
α-Farnesene	29.7	14	15	1	25.61	29.72	1499.51	3.63
Cyclohexene	29.81	15	16	1	29.72	33.64	1502.30	2.29
β-Sesquiphellandrene	30.44	15	16	1	29.72	33.64	1518.37	4.57

n, number of alkane carbons eluting before "i"; m, number of alkane carbons eluting after "i"; Dn, "m"-"n"; tri, retention time of i (sample); trn, retention time of standard "n" (standard before); trm, retention time of pattern "m" (pattern after); IKA, 100*n + 100*Dn* (trA-trn/trm-trn); %, Percent of the compound.

**Figure 4.**
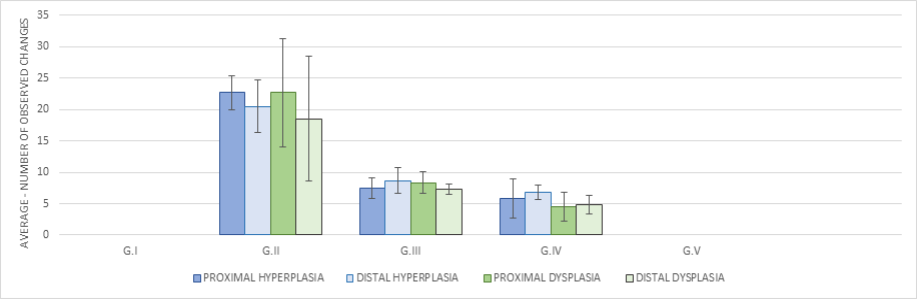
Number of Hyperplasias and Dysplasias Observed after HE Staining in the Proximal and Distal Portions of Each Group. The mean and standard deviation were calculated based on the number of animals in each group. Results were expressed as the mean of the amount observed in the animals of the same group ± standard deviation. * Significance per Tukey test p<0.05

**Table 2.** Averages of Aberrant Crypts (AC) and Aberrant Crypt Foci (ACF), in the Proximal and Distal Regions of the Colon, in Experimental Groups of Induction with DMH (1,2 dimethylhydrazine) and Essential Oil Treatment Zingiber Officinale and 5-FU

	Proximal Colon				Distal Colon			
	CA		FCA		CA		FCA	
	1 crypt	2-3 crypts	4-9 crypts	≥10 crypts	1 crypt	2-3 crypts	4-9 crypts	≥10 crypts
GI	0.25 ±0.5	0.00±0.0	0.00±0.0	0.00±0.0	0.00±0.0	0.00±0.0	0.75±1.5	0.00±0.0
GII	42.67±16.20	36.83±16.95	4.83±2.63	0.17±0.40	37.50±18.29	47.00±15.63	33.33±15.16	0.50±0.83
GIII	40.17±22.28^b^	34.17±12.44^b^	1.33±1.75^a^	0.00±0.0	18.50±11.94^a,b^	28.83±13.31^a^	16.67±10.55^a^	0.50±0.83
GIV	8.17±5.67	8.17±5.03	2.33±2.06	0.00±0.0	7.83±3.06	16.00±7.72	16.67±8.47	0.50±0.83
GV	0.00±0.0	0.00±0.0	0.00±0.0	0.00±0.0	0.00±0.0	0.00±0.0	0.00±0.0	0.00±0.0

AC, aberrant crypts; ACF, Aberrant Crypt Foci; GI, control group; GII, experimental group with DMH induction; GIII, experimental group with DMH induction + essential oil; GIV, experimental group with DMH + 5-FU induction; GV, experimental group with essential oil. Results expressed as average of the quantities observed in the animals of the same group ± standard deviation. ^a^, Significant when compared to GII; ^b^, Significant when compared to GIV. Significance when p<0.05.

## Discussion

This study was conducted through a bioassay with ACF to investigate whether orally administered GEO, after induction with carcinogen, can modify colon carcinogenesis in rats. 

Giada and Filho (2006) reported that compounds with antioxidant activity could affect the process of carcinogenesis in molecular events at all stages of the process, resulting in a reduction of potential cancer risk. Such compounds could inactivate the reactive oxygen and nitrogen species that play an important role in carcinogenesis, preventing the triggering of the process commonly catalysed by cytochrome P-450 enzymes. The antioxidant activity of ginger essential oil, according to the study by El-Ghorab et al., (2010), is related to the presence of antioxidant compounds such as camphene, p-cineol, borneol, R-terpineol and zingiberene, whose use showed a reduction of more than 50% in the activity of the free radical DPPH (2.2-Diphenyl-1-picryl-hydrazyl) at a 200 μg/ml concentration. 

Among the most important compounds present in our GEO sample, the monoterpenes citral, δ-citral and camphene show important pharmacological properties, including antioxidant, analgesic and antitumor activity (Souza et al., 2018). Our results are comparable to the literature results where the most significant phytochemical components found corroborate the study of Höferl et al., (2015), varying only the percentages (citral - 10.5%; camphene - 7.8%; α-zingiberene - 17.4%). Nevertheless, our results contrast with the study by Andrade et al., (2012) whose oil showed as major constituents the oxygenated monoterpenes geranial, neral, 1.8-cineole, geraniol, geranyl acetate and the bicyclic monoterpene, camphene. These differences may occur due to different climatic conditions and the place of origin of the samples, which ends up affecting their chemical composition. Given these results, we can infer that our GEO, for presenting a mixture of monoterpene and sesquiterpene compounds, has good antioxidant potential, capable of performing pharmacological action as described in the literature. 

The positive variation of the animals weight gain presented in Figure 1 demonstrates that the GEO does not compromise the intestinal absorption. On the other hand, when we compare GII and III, the general results of the number of ACF in both regions show that the GEO was significantly effective, inhibiting the clonal expansion of cells that make up the ACF. Similar to the results found with isolated AC, the ACF with the highest number of crypts in the distal GIII region had similar values to the GIV. This fact leads to the hypothesis that experimental treatment with GEO, despite not fully regulating the appearance of AC and ACF, exerted a chemoprotective action, and controlled the multiplicity of crypts/focus. 

In the analyses with HE staining, hyperplastic ACF and dysplastic ACF were visualized only in GII, GIII and GIV, with significantly higher values in GII when compared to GIII. Again, these results suggest that the GEO was effective in controlling AC clonal expansion in ACF. 

The results obtained are of great interest, as previous studies have already found that epithelial cells suffer from CRC pathogenesis from aberrant crypt, as this region favors the formation of malignant tumours, with higher numbers of ACF. In addition, ACF with increasing crypt multiplicity are more resistant to apoptotic cell death (Ansil et al., 2013; Kilari et al., 2016). In our study, no tumors were observed in any experimental group probably due to the duration of the experiment. According to the literature, the development of more advanced lesions occurs from the 22^nd^ week of exposure to carcinogen (Bird, 1987).

Yamashita et al., (1994) report that PCNA is a very useful marker to evaluate cell proliferative activity in ACF. The results of this study point to positively marked cells for PCNA antibody, mainly in the basal region of normal crypts. However, some cells in the upper portion of ACF were also marked. According to the authors, most of these cells were probably in the G1 phase of the cell cycle. At this cycle phase, cells suffer more rapidly division, especially when subjected to cell multiplication stimulators. In the MI evaluation of our results, we observed a clear increased of cells proliferation that make up the ACF, also in dysplastic ACF there was positive marking for PCNA in the basal region and in the upper portion of the ACF (Figure 3), in all experimental groups.

In this sense, even though the GEO does not present a significant reduction in MI values among the experimental groups, we can conclude that it has been able to control cell proliferation. The probable antioxidant activity performed by it is important in neutralizing free radicals produced at the moment of cell damaging stimulus that stimulates cell renewal/proliferation in mucosae.

In conclusion, since cell proliferation is necessary to fix genetic alterations during carcinogenesis, our findings suggest that ACF are regions with higher probability of developing neoplasms. Therefore, they constitute excellent biomarkers in bioassays for chemopreventive evaluation of compounds with probable antineoplastic activity. Furthermore, the assessment of the predictive value of neoplasm precursor lesions is increasingly necessary for treatments evaluation or cancer preventive measures (Rodrigues et al., 2002).

## References

[B1] Adams RP (2007). Identification of essential oil components by gas chromatography/mass spectrometry.

[B2] Andrade MA, Cardoso MG, Batista LR, Mallet ACT, Machado SMF (2012). Essential oils of Cymbopogonnardus, Cinnamomumzeylanicum and Zingiber officinale: composition, antioxidant and antibacterial activities. Rev Ciênc Agron.

[B3] Ansil PN, Prabha SP, Nitha A, Latha MS (2013). Chemopreventive effect of Amorphophalluscampanulatus (Roxb) blume tuber against aberrant crypt foci and cell proliferation in 1, 2-dimethylhydrazine induced colon carcinogenesis. Asian Pac J Cancer Prev.

[B4] Barreiros ALBS, David JM, David JP (2006). Oxidative Stress: Relationship Between Generation Of Reactive Species And Defense Of The Organism. New Chemistry.

[B5] Blanco EZ, Bajay MM, Siqueira MVBM, Zucchi MI, Pinheiro JB (2016). Genetic diversity and structure of Brazilian ginger germplasm (Zingiber officinale) revealed by AFLP markers. Genetica.

[B6] Bird RP (1987). Observation and qualification of aberrant crypt in the murine colon treated with a colon carcinogen: Preliminary findings. Cancer Lett.

[B7] Bird RP (1995). Role of aberrant crypt foci in understanding the pathogenesis of colon cancer. Cancer Lett.

[B8] Bird RP, Good CK (2000). The significance of aberrant crypt foci in understanding the pathogenesis of colon cancer. Toxicol Lett.

[B9] Cheng L, Lai MD (2003). Aberrant crypt foci as microscopic precursors of colorectal cancer. World J Gastroenterol.

[B10] Dias MC, Spinardi-Barbisan ALT, Rodrigues MA (2010). Lack of chemopreventive effects of ginger on coloncarcinogenesis induced by 1,2-dimethylhydrazine in rats. Food Chem Toxicol.

[B11] Dutra VGP, Parreira VAG, Guimarães RP (2018). Evolution of mortality for colorectal cancer in Brazil and regions, by sex, 1996-2015. Arq Gastroenterol.

[B12] Eisenberg ALA, Koifman S (2001). Breast cancer: tumor markers (Literature review). Rev Bra Cancerol.

[B13] El-Ghorab AH, Nauman M, Anjum FM, Hussain S, Nadeem M (2010). A comparative study on chemical composition and antioxidant activity of ginger (Zingiber officinale) and cumin (Cuminumcyminum). J Agricul Food Chem.

[B14] Eser S, Chang J, Charalambous H (2018). Incidence patterns of colorectal cancers in four countries of the middle east cancer consortium (Cyprus, Jordan, Israel, And Izmir, Turkey) compared with those in the united states surveillance, epidemiology, and end results program. Turk J Gastroenterol.

[B15] Fernandes RVB, Botrel DA, Silva EK (2016). Cashew gum and inulin: New alternative for ginger essential oil microencapsulation. Carbohydr Polymers.

[B16] Ganaie MA, Saeedan AAl, Madhkali H (2019). Chemopreventive efficacy zingerone (4-[4-hydroxy-3-methylphenyl] butan-2-one) in experimental colon carcinogenesis in Wistar rats. Environ Toxicol.

[B17] Giada MLR, Mancini Filho J (2006). Importance of dietary phenolic compounds in promoting human health. Publication UEPG: Cienc Biol Health.

[B18] Höferl M, Stoilova I, Wanner J (2015). Composition and comprehensive antioxidant activity of ginger (Zingiber officinale) essential oil from Ecuador. Nat Prod Commun.

[B19] Kilari BP, Kotakadi VS, Penchalaneni J (2016). Anti-proliferative and apoptotic effects of Basellarubra (L) against 1, 2-Dimethyl hydrazine-induced colon carcinogenesis in rats. Asian Pac J Cancer Prev.

[B20] Mendonça R, Valadão M, Couto AC, Koifan S (2012). Colorectal cancer mortality trend in five Brazilian capitals from 1980 to 2009. Public Health Notebook.

[B21] Moati E, Taly V, Didelot A (2018). Role of circulating tumor DNA in the management of patients with colorectal cancer. Clin Res Hepatol Gastroenterol.

[B22] Moghadamtousi SZ, Fadaeinasab M, Nikzad S (2015). Annona muricata (Annonaceae): A review of its traditional uses, isolated acetogenins and biological activities. Int J Mol Sci.

[B23] Pan MH, Lai CS, Wu JC, Ho CT (2011). Molecular mechanisms for chemoprevention of colorectal cancer by natural dietary compounds. Mol Nutr Food Res.

[B24] Raju J (2008). Azoxymethane-induced rat aberrant crypt foci: Relevance in studying chemoprevention of colon cancer. World J Gastroenterol.

[B25] Rodrigues MAM, Silva LAG, Salvadori DMF, De Camargo JLV, Montenegro MR (2002). Aberrant crypt foci and colon cancer: comparison between a short- and medium term bioassay for colon medium-term bioassay for colon carcinogenesis using dimethylhydrazine in Wistar rats. Braz J Med Biol Res.

[B26] Scopa CD, Tsamandas AC, Zolota V (2003). Potential role of bcl-2 and Ki-67 expression and apoptosis in colorectal carcinoma A clinicopathologic study. Dig Dis Sci.

[B27] Sharma JN, Srivastava KC, Gan EK (1994). Suppressive effects of eugenol and ginger oil on arthritic rats. Pharmacology.

[B28] Souza MRP, Coelho NP, Baldin VP (2018). Synthesis of novel (-)-Camphene-based thiosemicarbazones and evaluation of anti-Mycobacterium tuberculosis activity. Nat Prod Res.

[B29] Tanaka T (2009). Colorectal carcinogenesis: Review of human and experimental animal studies. J Carcinog.

[B30] Togar B, Türkez H, Stefano A, Tatar A, Cetin D (2014). Zingiberene attenuates hydrogen peroxide-induced toxicity in neuronal cells. Hum Exp Toxicol.

[B31] Valadão M, Castro LS (2018). Hereditary colorectal cancer. Rev Col Bras Cirurg.

[B32] Voon CH, Bhat R, Rusul G (2012). Flower extracts and their essential oils as potential antimicrobial agents for food uses and pharmaceutical applications. Compr Rev Food Sci Food Safety.

[B33] Yoshimi N, Morioka T, Kinjo T (2004). Histological and immunohistochemical observationsof mucin-depleted foci (MDF) stained with Alcian blue, in rat coloncarcinogenesis induced with 1,2-dimethylhydrazine dihydrochloride. Cancer Sci.

[B34] Yamashita N, Minamoto T, Onda M, Esumi H (1994). Increased cell proliferation of azoxymethane-induced aberrant crypt foci of rat colon. Jpn J Cancer Res.

